# Dietary olive oil effect on antioxidant status and fatty acid profile in the erythrocyte of 2,4-D- exposed rats

**DOI:** 10.1186/1476-511X-9-89

**Published:** 2010-08-25

**Authors:** Amel Nakbi, Wafa Tayeb, Samia Dabbou, Manel Issaoui, Abir K Grissa, Nabil Attia, Mohamed Hammami

**Affiliations:** 1 Biochemistry Laboratory, UR03/ES08 'Human Nutrition & Metabolic Disorders', USCR Mass Spectrometry, Faculty of Medicine of Monastir, Tunisia; 2 King Saud University, Riyadh, Saudi Arabia

## Abstract

**Background:**

Oxidative stress produced by reactive oxygen species (ROS) has been linked to the development of several diseases such as cardiovascular, cancer, and neurodegenerative diseases. This study investigates the possible protective effect of extra virgin olive oil (EVOO), lipophilic fraction (OOLF) and hydrophilic fraction (OOHF) on oxidative stress and fatty acid profile of erythrocytes in 2,4-D treated rats.

**Methods:**

Male Wistar rats were divided randomly into eight groups: control (C), (2,4-D) at a dose of 5 mg/kg b.w., (2,4-D/EVOO) was given 2,4-D plus EVOO, (2,4-D/OOHF) that received 2,4-D plus hydrophilic fraction, (2,4-D/OOLF) treated with 2,4-D plus lipophilic fraction, (EVOO) that received only EVOO, (OOHF) was given hydrophilic fraction and (OOLF) treated with lipophilic fraction. These components were daily administered by gavages for 4 weeks.

**Results:**

2,4-D treatment lead to decrease of antioxidant enzyme activities, namely, superoxide dismutase (SOD), catalase (CAT), glutathione peroxidase (GPx) and glutathione reductase (GR) associated with a higher amount of MDA level. Erythrocyte membranes' fatty acid composition was also significantly modified with 2,4-D exposure. EVOO and hydrophilic fraction supplemented to rats with or not 2,4-D treatment enhanced the antioxidant enzyme activities and reduced the MDA level. However, lipophilic fraction did not show any improvement in oxidative damage induced by 2,4-D in spite its richness in MUFA and vitamins.

**Conclusion:**

EVOO administered to 2,4-D-treated rats protected erythrocyte membranes against oxidative damage by means of preventing excessive lipid peroxidation to increase the MUFA composition and increase maintaining antioxidants enzymes at normal concentrations.

## Background

Oxidative stress produced by free radicals has been linked to the development of several diseases such as cardiovascular, cancer, and neurodegenerative diseases [1]. However, reactive oxygen species (ROS) are constantly formed as by-products of normal metabolic reactions and their formation is accelerated by accidental exposure to occupational chemicals like pesticides. Since 2,4-D (2,4-dichlorophenoxyacetic acid) is a common herbicide used around the home and garden, on golf courses, ball fields, parks, in agriculture and forestry. Several reports have shown that 2, 4-D produces oxidative stress and/or depletes antioxidants both *in vitro *and *in vivo*. *In vitro*, studies have mainly dealt with the effect of the herbicide on hepatocytes and red blood cells [2-6], while *in vivo *oxidative activity has been proved in different species including yeast [7,8], fish [9,10] and rats [11].

Recently, there is growing evidence that ROS contribute to organ injury in many systems including heart, liver and central nervous system [12]. Erythrocytes are permanently in contact with potentially damaging levels of oxygen, but their metabolic activity is capable of reversing this injury under normal conditions. Erythrocytes are equipped by many defence systems representing their antioxidant capacity [13]. This protective system includes superoxide dismutase (SOD), catalase (CAT), reduced glutathione, glutathione peroxidase (GPx), glutathione-S-transferase, and glutathione reductase (GR). However, the cellular antioxidant action is reinforced by the presence of dietary antioxidants.

Olive oil is the main source of fat in the Mediterranean diet, which has been shown to be effective against oxidative stress associated diseases. It has been reported also that olive oil is able to reduce the risk of coronary heart disease (CHD) by decreasing levels of artery-clogging lipids in the blood [14]. Other Studies have shown that olive oil offers protection against heart disease by controlling LDL ("bad" cholesterol) levels while raising HDL (the "good" cholesterol) levels [15]. In fact, the beneficial effects of olive oil on CHD risk have been attributed to its high monounsaturated fatty acid (MUFA) content, mostly in the form of oleic acid (18:1n-9), which ranges from 70 to 80% of total fatty acids [16]. Nevertheless, evidences have accumulated on the beneficial properties of minor though highly bioactive components of olive oil [17,18].

MUFA-enriched diets have shown no long-term ill effects and are associated with reduced rates of CHD. Furthermore, replacement of saturated fatty acids (SFAs) with MUFA-enriched diets appears to have beneficial effects on lipoprotein concentrations in both diabetic [19] and nondiabetic persons [20]. Moreover, in Nonalcoholic Fatty Liver Disease (NAFLD), exposure of murine or human hepatocytes to MUFA resulted in lipid accumulation without changes in cell viability, while cell incubation with SFAs significantly decreased cell viability and increased caspase activation and apoptosis [21].

Healthy effects of dietary MUFA were also attributed to decreased endothelial activation [22,23] and tendency of LDL to oxidation [24]. Likewise, epidemiological studies showed an inverse correlation between the intake of long-chain n-3 polyunsaturated fatty acids (PUFA) present in fish and fish oil and the incidence of CHD [25]. However, there are controversial reports on the cellular and molecular mechanisms involved in these preventive effects. Previous studies [26,27] have shown that enrichment of the diet with MUFA at the expense of PUFA led to LDLs that were less susceptible to oxidation, as determined by *in vitro *assays. In fact, further studies *in vitro *suggested that lipoproteins enriched with oleic acid are less susceptible to oxidation than those enriched with linoleic or arachidonic acid and may have less proinflammatory activity when exposed to oxidizing conditions [28]. Hence, oxidative stress is a process that mainly affects structural lipids especially PUFA. Furthermore, oxidative stress could play an important role in the modulation of the liver fatty acid desaturase activity [29]. Recent studies showed that hepatic lipotoxicity include abnormal fatty acid oxidation with formation of ROS and disturbances in cellular membrane fatty acid and phospholipid composition [30]. Limited evidence from animal studies suggests that MUFA-rich membranes are more resistant to oxidative processes, protecting the aged cell [31,32]. Moreover, mitochondrial structure, integrity and DNA stability to oxidation were also enhanced when rats consumed a extra virgin olive oil (EVOO) rich diet [32].

The beneficial effects of olive oil in the Mediterranean diet can be attributed not only to the close relationship between unsaturated and saturated fatty acids, but also to the antioxidant property of its phenolic compounds. So, the main antioxidants of EVOO are carotenoids and phenolic compounds, which are both lipophilic and hydrophilic. The lipophilics include tocopherols, while the hydrophilics include flavonoids, phenolic alcohols and acids, secoiridoids and their metabolites. However, the antioxidant properties of the phenolic compounds of olive oil have been the most extensively studied. In experimental studies, olive oil phenolic compounds, showed strong antioxidant properties against lipids, DNA, and LDL oxidation [33,34]. In animal models, olive oil phenolics retained their antioxidant properties *in vivo *[35] and delayed the progression of atherosclerosis [36]. Its other ways as result evident that β-sitosterol and the polyphenols in olive oil (oleuropein, tyrosol, hydroxytyrosol, and caffeic acid) inhibit the formation of oxygen reactive species [37,38], reduce the susceptibility of LDL oxidation, erythrocyte membranes to lipid peroxidation [39,40], and increase the lag time of conjugated diene formation in a concentration-dependent manner [41]. Hence, most of these studies have been performed *in vitro *and literature data on olive oil polyphenols mainly concern purified compounds. While the total fraction antioxidant properties of the lipophilic or hydrophilic components has been poorly investigated. Being a complex mixture of compounds, the measure of the total antioxidant capacity could be more representative than the protective effect of a single component.

Thus, the aim of this study was to investigate the effects of extra virgin olive oil and its lipophilic and hydrophilic fractions on oxidative stress and fatty acid composition of erythrocytes in 2,4-D treated rats.

## Results

### Analytical parameters of extra virgin olive oil and its compounds

The analytical parameters fatty acid, oxidative stability and antioxidant composition of the olive oils employed is shown in Table 1. It can be seen that the phenolic compounds were efficiently removed from EVOO, being much reduced in the olive oil lipophilic fraction (OOLF). The procedure employed to produce the OOLF, at variance with a washing process, has been developed to eliminate selectively hydrophilic substances, such as phenolic compounds, leaving the other olive oil components unmodified such as tocopherols and fatty acids (Table 1). While, olive oil hydrophilic fraction (OOHF) contained only phenolic compounds (384 mg/kg).

**Table 1:** Mean values of analytical parameters, fatty acids composition (%), oxidative stability and antioxidant content of extra virgin olive oil (EVOO), hydrophilic fraction (OOHF), lipophilic fraction (OOLF) and standart diet fed to rat

	Extra virgin olive oil (EVOO)	Lipophilic fraction (OOLF)	Hydrophilic fraction (OOHF)	Standard diet
Palmitic acid [%]	10.28 ± 0.04^a^	10.40 ± 0.01^a^	-	11.62 ± 0.85^a^
Palmitoleic acid	0.77 ± 0.30^a^	0.79 ± 0.60^a^	-	0.10 ± 0.04 ^a^
Stearic acid	3.39 ± 0.14^a^	3.56 ± 0.37^a^	-	1.15 ± 0.32 ^b^
Oleic acid	64.80 ± 1.99 ^a^	62.58 ± 3.71 ^a^	-	29.73 ± 1.12 ^b^
Linoleic acid	14.34 ± 0.90 ^b^	15.04 ± 0.54 ^b^	-	53.89 ± 1.49^a^
Linolenic acid	0.64 ± 0.04^a^	0.68 ± 0.03 ^a^	-	0.61 ± 0.02 ^a^
Arachidic acid	0.74 ± 0.05^a^	0.78 ± 0.02^a^	-	0.09 ± 0.04 ^b^
Gadoleic acid	0.62 ± 0.03^a^	0.57 ± 0.03^a^	-	0.14 ± 0.04 ^b^
Behenic acid	2.84 ± 0.37^a^	2.87 ± 0.80^a^	-	0.22 ± 0.10 ^b^
SFA	17.28 ± 0.22^a^	17.62 ± 0.42 ^a^	-	13.08 ± 0.21 ^b^
MUFA	66.20 ± 2.34 ^a^	63.95 ± 3.07 ^a^	-	29.97 ± 1.32 ^b^
PUFA	14.99 ± 0.94^b^	15.72 ± 0.57^b^	-	54.50 ± 2.12^a^
MUFA/PUFA	4.43 ± 0.43 ^a^	4.07 ± 0.04^a^	-	0.54 ± 0.32 ^b^

OSI (h)	9.6^a^	4.7 ^b^	-	-
Chlorophylls (mg/kg)	12.65 ± 0.29^a^	5.4 ± 0.76 ^b^	-	ND
β-Carotene (mg/kg)	7.15 ± 0.20 ^a^	5.43 ± 0.08 ^b^	-	ND
Total polyphenols (mg/kg)*	579.17 ± 71.40^a^	-	384 ± 23.18 ^b^	ND
α- tocopherol (mg/kg)	484.56 ± 11.11^a^	491.64 ± 10.36^a^	-	224 ± 11.86 ^b^

Data are expressed by mean values ± of three independent experiments. SFA: saturated fatty acids; MUFA: monounsaturated fatty acid; PUFA: polyunsaturated fatty acid; OSI: oxidative stability Index. ND: indicates not determined

Values followed by same letters are not significantly different. (Tukey's test, *p *< 0.05)

* As hydroxytyrosol equivalents (mg/kg EVOO)

### Erythrocyte oxidative damage

All the results from various treatment groups were compared with their normal control (C). However, results from D/EVOO, D/OOHF or D/OOLF groups were also compared with the data of 2,4-D treated group (D). During this study, no sign of toxicity was observed under 2, 4-D treatment at 5 mg/kg. Rats exhibited normal behavior in comparison to the control group. There were no significant differences between tested and control groups of rats in body weight gain during the experiment (Table 2).

**Table 2:** Growth parameters of control and treated rats

	Initial body weight (g)	Final body weight (g)	Weight gain (%)
**C**	216.80 ± 18.10	273.70 ± 27.10	26.16 ± 4.05
**D**	220.00 ± 11.11	276.70 ± 15.23	25.62 ± 5.66
**D/EVOO**	222.80 ± 19.03	280.60 ± 19.77	26.21 ± 6.15
**D/OOHF**	222.60 ± 25.29	279.10 ± 34.74	25.32 ± 5.67
**D/OOLF**	219.10 ± 16.17	277.40 ± 21.66	26.69 ± 5.90
**EVOO**	221.30 ± 22.84	271.10 ± 22.96	22.81 ± 6.10
**OOHF**	225.50 ± 23.10	286.10 ± 25.86	27.08 ± 4.33
**OOLF**	221.50 ± 30.64	284.50 ± 29.53	29.82 ± 6.85

Data are expressed as means ± SD (n = 10 rats per group). C: controls group, D: 2, 4-D treated group, D/EVOO: 2, 4-D plus extra virgin olive oil, D/OOHF: .2, 4-D plus hydrophilic fraction, D/OOLF: .2, 4-D plus lipophilic fraction, EVOO: extra virgin olive oil treated group alone, OOHF: group treated with hydrophilic fraction of olive oil alone, OOLF: group treated with lipophilic fraction of olive oil alone. Comparison between groups was made using ANOVA one way analysis with Bonferroni test.

**Figure 1:**
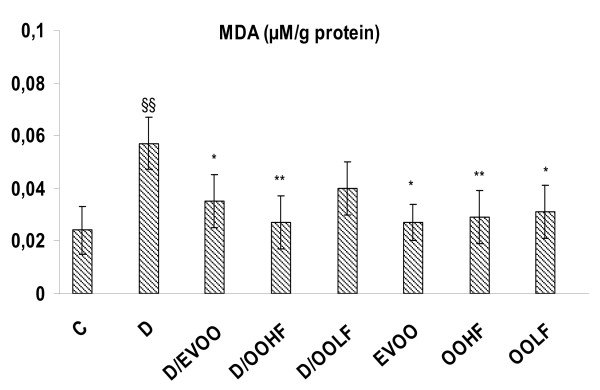
**Plasma MDA level in the treatment groups for 4 weeks**. C: controls group, D: 2,4-D treated group at dose 5 mg/kg BW, D/EVOO: 2,4-D (5 mg/kg BW) plus extra virgin olive oil, D/OOHF: .2,4-D plus hydrophilic fraction, D/OOLF: .2,4-D plus lipophilic fraction, EVOO: extra virgin olive oil treated group alone, OOHF: group treated with hydrophilic fraction of olive oil, OOLF: group treated with lipophilic fraction of olive oil. Data are expressed as means ± SD (n = 10 rats per group). Comparison between groups was made using ANOVA with Bonferroni correction (* *p *< 0.05; ** *p *< 0.01 compared to Group D) (^§§ ^*p *< 0.01 compared to controls C).

Figure 1 shows that plasma MDA level was doubled in 2,4-D treated group compared to control group. EVOO and OOHF administrated to rats treated at the same time with 2,4-D alleviated lipid peroxidation induced by the herbicide and modulated significantly the level of MDA in plasma (*p *< 0.05 and *p *< 0.01, respectively). While lipophilic extract supplemented to 2,4-D treated rats showed less improvement in the level of MDA induced by the herbicide. EVOO, OOHF and OOLF treatment alone did not result in significant changes in the level of MDA compared to control group.

As for the evaluation of antioxidant enzyme activities, our results revealed that 2,4-D caused a statistically significant decrease in SOD, GPx, GR and CAT activities in rat erythrocytes (*p *≤ 0.05) (Table 3). The addition of EVOO and OOHF to 2,4-D treated group normalized the levels of these antioxidant enzymes. Whereas, OOLF administration to 2,4-D treated animals led to a statistically significant decrease in SOD, GPx, GR and CAT activities (*p *< 0.05), compared to the control value. The change in the activities accounted to 36%, 47%, 41% and 16%, respectively, in rats erythrocytes. Treatment with EVOO and OOHF alone did not provoke a significant alteration in the antioxidant enzyme activities compared to control treatment, except for CAT activity which was significantly increased after OOHF administration (*p *< 0.05). Furthermore, alone OOLF supplementation to rat, induced a significant reduction in the SOD, GPx, and GR activities (*p *< 0.05) and the change accounted to 22% (*p *< 0.05), 52% (*p *< 0.01) and 28% (*p *< 0.05) respectively, compared to the control values (Table 3).

**Table 3:** Erythrocytes antioxidants enzymes activities in control and experimental groups

Groups	Antioxidant enzyme activity (U/g Hg)			
	
	SOD	GPx	GR	CAT
**C**	644.89 ± 28.41	489.05 ± 24.86	6.11 ± 0.40	1237.27 ± 46.48
**D**	598.54 ± 18.62^§^	404.22 ± 21.72^§^	3.69 ± 1.41^§^	1093.22 ± 64.20^§^
**D/EVOO**	641.56 ± 14.32	485.97 ± 20.56*	5.23 ± 0.84	1368.077 ± 113.67**
**D/OOHF**	623.75 ± 71.43	460.18 ± 21.07	5.41 ± 0.69	1210.71 ± 53.11
**D/OOLF**	415.07 ± 94.91**^§§^	259.18 ± 25.74^§§^*	3.62 ± 0.76^§^	1044.30 ± 47.37^§§^
**EVOO**	655.47 ± 24.74	476.91 ± 33.58	5.98 ± 0.68*	1180.51 ± 53.82
**OOHF**	633.78 ± 29.42	472.49 ± 49.48	5.51 ± 0.75	1373.96 ± 71.51**
**OOLF**	508.52 ± 80.23^§^	235.27 ± 48.06^§§^*	4.42 ± 1.00^§^	1162.88 ± 85.58

Data are means ± SD; n = 10 for all groups. C: controls group, D: 2, 4-D treated group, D/EVOO: 2, 4-D plus extra virgin olive oil, D/OOHF: .2, 4-D plus hydrophilic fraction, D/OOLF: .2, 4-D plus lipophilic fraction, EVOO: extra virgin olive oil treated group alone, OOHF: group treated with hydrophilic fraction of olive oil, OOLF: group treated with lipophilic fraction of olive oil. SOD: Superoxide dismutase, GPx: Glutathione peroxidase, GR: Glutathione reductase, CAT: catalase.

Comparison between groups was made using ANOVA one way analysis with *Bonferroni test*.

** p <.01; * p < .05 (compared to Group D)

^§§ ^p < .01; ^§ ^p < .05 (compared to controls C).

### Fatty acid composition of erythrocyte membranes

The fatty acid composition of erythrocyte membranes reflects dietary lipid composition. The changes in erythrocyte membranes fatty acids are given in Table 4. Rats fed a standard diet with 2,4-D tended to have higher total SFA proportions in erythrocyte than control rats, and caused by a significant rise of 18:0, 20:0 and 22:0. Whereas total MUFA level was decreased by a significant decline of C22:1 and C24:1 (*p *< 0.05). The proportion of PUFA in total lipids of erythrocyte was somewhat lowered in 2,4-D treated animals, caused by a reduction of 20:2 (n-6), 22:4 (n-6), 22:5 (n-3) and 22:6(n-3). Consequently, a significant decrease of PUFA/SFA and UFA/SFA ratios was observed in erythrocyte membranes of 2,4-D- treated rats.

**Table 4:** Erythrocyte membranes fatty acid composition of control and treated rats

FA%	C	D	D/EVOO	D/OOHF	D/OOLF	EVOO	OOHF	OOLF
C14:0	2.30 ± 0.36	2.19 ± 0.49	2.08 ± 0.34	2.18 ± 0.33	2.26 ± 0.43	2.23 ± 0.34	1.94 ± 0.28	1.93 ± 0.30
C16:0	25.96 ± 1.32	26.44 ± 1.90	22.34 ± 1.71^§§^**	25.15 ± 1.89	24.17 ± 1.87	20.31 ± 1.15^§§^**	24.41 ± 1.68	20.91 ± 1.74^§§^**
C18:0	16.54 ± 3.30	22.96 ± 1.26^§^	19.78 ± 1.89*	20.94 ± 0.96*	20.58 ± 2.03*	15.37 ± 1.34**	19.27 ± 1.24**	20.80 ± 3.50*
C20:0	0.20 ± 0.04	0.36 ± 0.09^§§^	0.21 ± 0.06**	0.19 ± 0.08**	0.41 ± 0.08^§§^	0.22 ± 0.05**	0.21 ± 0.12**	0.39 ± 0.05^§§^
C22:0	1.04 ± 0.24	1.65 ± 0.47^§^	0.86 ± 0.21**	1.21 ± 0.32	1.24 ± 0.40	1.14 ± 0.26	1.53 ± 0.38	1.16 ± 0.15
C24:0	0.94 ± 0.26	1.11 ± 0.36	0.92 ± 0.37	0.54 ± 0.21*	1.13 ± 0.44	0.95 ± 0.42	0.47 ± 0.21^§^**	1.12 ± 0.63
**ΣSFA**	**46.98 ± 4.48**	**54.71 ± 3.26**^§^	46.19 ± 4.49*	50.21 ± 3.75*	**49.79 ± 3.32**	**40.22 ± 2.69**^§^**	47.83 ± 3.76*	46.31 ± 4.05*
C14:1	0.55 ± 0.14	0.71 ± 0.15	0.76 ± 0.16	0.74 ± 0.11	0.54 ± 0.12	0.73 ± 0.09	0.55 ± 0.12	0.64 ± 0.10
C16:1	0.40 ± 0.06	0.33 ± 0.07	0.63 ± 0.07^§^**	0.53 ± 0.09*	0.38 ± 0.05	0.57 ± 0.05^§^**	0.51 ± 0.05**	0.34 ± 0.06
C18:1	17.15 ± 0.19	15.67 ± 1.10	19.64 ± 1.33*	15.54 ± 1.17	16.08 ± 1.35	21.88 ± 0.96^§§^**	17.74 ± 0.37	19.30 ± 1.14^§^*
C20:1	0.23 ± 0.07	0.20 ± 0.06	1.06 ± 0.18^§§^**	0.40 ± 0.12^§^*	0.22 ± 0.08	0.42 ± 0.12^§^*	1.35 ± 0.40^§§^**	0.38 ± 0.05^§^*
C22:1	0.18 ± 0.04	0.09 ± 0.03^§^	1.13 ± 0.23^§^	0.46 ± 0.11^§§^	0.20 ± 0.03**	0.87 ± 0.24	1.5 ± 0.24^§§^**	1.67 ± 0.56^§^*
C24:1	1.62 ± 0.16	0.89 ± 0.25^§^	1.97 ± 0.56*	1.44 ± 0.39	1.37 ± 0.39	2.27 ± 0.46*	1.27 ± 0.28	1.66 ± 0.73
**ΣMUFA**	**20.13 ± 2.89**	**17.89 ± 0.66**^§^	**25.19 ± 0.40**^§^**	**19.11 ± 1.60**	**18.79 ± 1.76**	26.74 ± 1.28**^§§^	22.92 ± 0.65**	**23.99 ± 1.63**^§^**
C18:2 *ω*6	11.76 ± 0.84	11.19 ± 0.82	11.41 ± 0.74	11.53 ± 0.48	11.50 ± 0.21	11.58 ± 0.66	11.83 ± 1.32	11.44 ± 0.39
C18:3w3ALA	0.36 ± 0.09	0.39 ± 0.1	0.48 ± 0.04	0.39 ± 0.05	0.49 ± 0.04	0.43 ± 0.09	0.40 ± 0.18	0.65 ± 0.14^§§^*
C20:2 *ω*6	0.29 ± 0.10	0.52 ± 0.20^§^	0.36 ± 0.13	0.30 ± 0.10*	0.79 ± 0.18^§§^*	0.36 ± 0.10	0.34 ± 0.11	0.57 ± 0.17^§§^
C20:3 *ω*6	0.40 ± 0.14	0.24 ± 0.07	0.41 ± 0.17	0.29 ± 0.10	0.60 ± 0.24*	0.56 ± 0.25*	0.52 ± 0.14*	0.63 ± 0.37*
C20:4 *ω*6	14.39 ± 2.03	14.45 ± 1.81	11.35 ± 1.70^§§^**	13.57 ± 1.85	12.81 ± 1.47	11.20 ± 1.48^§§^**	14.17 ± 1.82	12.76 ± 1.51
C20:5w3EPA	0.31 ± 0.16	0.35 ± 0.19	0.39 ± 0.02	0.46 ± 0.12	0.33 ± 0.14	0.73 ± 0.12^§§^*	0.63 ± 0.12^§§^*	0.73 ± 0.14^§§^*
C22:4 *ω*6	0.82 ± 0.19	0.32 ± 0.08^§§^	0.67 ± 0.05**	1.08 ± 0.15^§^**	1.21 ± 0.19^§^**	1.05 ± 0.30*	0.68 ± 0.16*	1.25 ± 0.23^§^**
C22:5w3DPA	0.67 ± 0.24	0.18 ± 0.07^§§^	0.80 ± 0.15**	0.30 ± 0.17^§^	0.23 ± 0.14^§^	1.03 ± 0.27^§^**	0.29 ± 0.16^§^	0.29 ± 0.15^§^
C22:6w3DHA	1.34 ± 0.31	0.20 ± 0.07^§§^	0.94 ± 0.20**	1.98 ± 0.46^§^**	1.48 ± 0.43**	1.84 ± 0.42**	1.36 ± 0.25**	1.05 ± 0.26**
**ΣPUFA**	**30.34 ± 3.14**	**27.84 ± 0.81**	**26.81 ± 1.93**^§^	**29.90 ± 1.85**	**29.44 ± 0.97**	**28.78 ± 0.75**	30.22 ± 1.73*	**29.37 ± 0.74**
**ΣUFA**	**41.77 ± 1.07**	**35.29 ± 0.64^§§^**	38.99 ± 1.49^§^*	39.96 ± 0.89^§§^**	**35.96 ± 1.77^§§^**	41.85 ± 1.41**	**36.95 ± 1.13^§§^**	44.22 ± 1.87**
PUFA/MUFA	1.50 ± 0.10	1.55 ± 0.11	1.06 ± 0.12^§§^**	1.56 ± 0.15	1.56 ± 0.13	1.07 ± 0.10^§§^**	1.31 ± 0.04	1.22 ± 0.07^§§^**
PUFA/SFA	0.64 ± 0.05	0.50 ± 0.05^§§^	0.58 ± 0.05*^§^	0.59 ± 0.03*	0.59 ± 0.04*	0.71 ± 0.03^§^**	0.63 ± 0.04**	0.63 ± 0.07**
UFA/SFA	0.88 ± 0.06	0.64 ± 0.05^§§^	0.84 ± 0.08*	0.79 ± 0.04	0.72 ± 0.10	1.04 ± 0.18^§^**	0.77 ± 0.08	0.95 ± 0.05**

Data are means ± SD; n = 10 for all groups. C: controls group, D: 2, 4-D treated group, D/EVOO: 2, 4-D plus extra virgin olive oil, D/OOHF: .2, 4-D plus hydrophilic fraction, D/OOLF: 2,4-D plus lipophilic fraction, EVOO: extra virgin olive oil treated group alone, OOHF: group treated with hydrophilic fraction of olive oil, OOLF: group treated with lipophilic fraction of olive oil.

SFA: Saturated fatty acid, MUFA: monounsaturated fatty acid, PUFA: polyunsaturated fatty acid, UFA: unsaturated fatty acid

** p <.01; *p < .05 (compared to Group D); ^§§ ^p < .01; ^§ ^p < .05 (compared to controls C)

Comparison between groups was made using ANOVA one way analysis with *Bonferroni test*

In erythrocyte membranes of rat supplemented EVOO with or without 2,4-D treatment had significantly lower total SFA levels and higher total MUFA proportions than controls and 2,4-D treated rats. This effect was mainly due an increase in 16:1, 18:1, 20:1 and 24:1 at the extent of 16:0, 18:0 and 20:0 (Table 4). Total lipids in erythrocytes from EVOO supplemented rats treated or not with 2,4-D tended to have a lower proportion of PUFA than those from controls and 2,4-D treated groups, which was due to a significant reduction in 20:4 (n-6), 22:4 (n-6), 22:5 (n-3) and 22:6 (n-3) levels. Furthermore, the saturation indexes (UFA/SFA) and (PUFA/SFA) were increased in erythrocyte membranes compared to 2,4-D treated group. While polyunsaturated to monounsaturated fatty acids (PUFA/MUFA) tended to be lowered over 4 weeks (Table 4).

No differences were found among hydrophilic and lipophilic fractions administered with or without 2,4-D groups for fatty acid composition of the erythrocyte membranes compared to controls except for the OOLF group. This latter group showed a significant raise of MUFA proportion. The (UFA/SFA) and (PUFA/SFA) ratios were somewhat elevated in erythrocyte membranes of rats supplemented hydrophilic or lipophilic fractions with or without 2,4-D treatment than that of controls and 2,4-D-treated rats whereas (PUFA/MUFA) ratio was decreased.

## Discussion

The present study investigates the potential protective effect of EVOO and its fractions (hydrophilic and lipophilic) supplementation to animals treated with 2,4-D pesticide. The increase of MDA level and the impairment of antioxidant enzyme activities clearly indicated that 2,4-D had the potency to cause oxidative damage in erythrocytes. In fact, it has been previously reported that acute exposure to 2,4-D may induce oxidative stress in rats [11]. The authors found that the administration of 1.5 and 3 mg/d of 2,4-D during 25 days might affect antioxidant potential enzymes, the activity of hepatic damage enzymes and lipid peroxidation. Other investigators have examined *in vitro *effects of 2,4-D on the generation of ROS, either at the mitochondrial level in hepatocytes or in red blood cells [3,6]. However, it has been shown that superoxide radical product can directly inhibit the activities of enzymes SOD, GPx and CAT, likewise, singlet oxygen and peroxyl radicals have been shown to inhibit SOD and CAT activities [42,43]. These observations may be explaining the significant decrease of SOD, GPx, GR and CAT activities in erythrocytes of 2,4-D-treated rats compared to controls. Furthermore, it was known that 2,4-dichlorophenol strongly decreases the level of reduced glutathione [44] and depletes the activity of the enzymes probably by disturbing their tertiary structure [5,45].

The co-administration of EVOO or OOHF counteracted the deleterious effects of 2,4-D by scavenging or neutralizing ROS. In fact, a marked decrease of MDA level and an increase of antioxidant enzyme activities were observed in EVOO and OOHF supplemented groups. However, despite its content in α- tocopherol, oral administration of OOLF with 2,4-D for 4 weeks did not show a protective role against oxidative stress caused by 2,4-D in erythrocyte rats. Therefore, we can suggest that the protective effects of EVOO are mostly attributed to the antiperoxidative property of its polar fraction. Indeed, *in vitro *and *ex vivo *models, olive oil phenolic compounds were proved to have antioxidant properties, higher than that of vitamin E, on lipids and DNA oxidation [46,47]. This study reported that olive oil phenolic compounds are also able to enhance the mRNA transcription of the antioxidant enzyme glutathione peroxidase. Furthermore, other studies have demonstrated a higher resistance to oxidation of LDL obtained from animals fed virgin olive oil, as compared to LDL separated from animals that were only administered an equivalent amount of oleic acid as either triolein [48] or dietary non-tocopherol antioxidants present in EVOO [49]. In our previous study, we found that EVOO with a higher phenolic content seemed to have beneficial effects on LDL particles oxidizability [50].

Moreover, it has been reported that phenolic compounds are able to interact with biological systems and to act as bioactive molecules; in particular they are important inhibitors of lipid peroxidation [51] and are believed to be effective through their free radical scavenging and metal chelating properties [52]. Several studies have demonstrated the ability of olive oil to inhibit oxidative stress in the liver through various mechanisms [53]. A study in our laboratory showed that oral supplementation of olive oil to rats administered ethanol chronically restored damage caused in the liver by inhibiting lipid peroxidation along with improving of the activities of enzymes [54].

The antioxidants enzymes (SOD, GPx, GR and CAT) limit the effects of oxidant molecules on tissues and are active in the defence against oxidative cell injury by means of their being free radical scavengers [55]. These enzymes work together to eliminate ROS where small deviations in physiological concentrations may have a dramatic effect on the resistance of cellular lipids, proteins and DNA to oxidative damage. Lipid peroxidation is the process of oxidative degradation of PUFA and its occurrence in biological membranes causes impaired membrane function, structural integrity, decrease in membrane fluidity and inactivation of a several membrane bound enzymes [56]. From the experiment described above, it is quite evident that the fatty acid composition of the erythrocyte membranes can be altered by 2,4-D exposure during 4 weeks. Knowing that PUFAs are more susceptible to peroxidation resulting in MDA formation in mammalian tissues [57] as a consequence a significant increase of SFA and decrease of MUFA and PUFA proportions were observed in erythrocyte membranes of 2,4-D treated rats. In fact, due to their peculiar structure, that is the presence of one or more double bonds, UFA are more susceptible to free radical damage. Consequently, a significant decrease in the PUFA/SFA and UFA/SFA ratios were seen in erythrocyte membranes of 2,4-D exposure rats. These results may explain the higher amounts of MDA observed in 2,4-D treated group.

Whereas, EVOO supplemented to rats treated or not with 2,4-D significantly reduced the proportion of SFA and enhanced the level of MUFA, mostly in the form of oleic acid, in erythrocyte membranes of rats. PUFA proportion was somewhat decreased compared to control group. Consequently, a lower amount of MDA was observed in these groups. This modification showed the protecting effects of the tested compounds supplemented diet which counteracted the deleterious effects of the herbicide, by their supplement in MUFA, phenolic compounds and vitamins. In fact, most of the studies comparing the effects of a MUFA-rich diet with PUFA-rich diet on LDL oxidation parameters have found a higher resistance of LDL particles to oxidation after the consumption of MUFA-rich diet [58]. Therefore the differences in erythrocyte membranes' fatty acid composition reflected the different fatty acid compositions of the diets and may be responsible for the differences in their tendency to lipid peroxidation.

## Conclusion

The results obtained from this study illustrated the oxidative damage and the changes of the fatty acid profile in erythrocyte membranes in rats resulting from subacute 2,4-D exposure. The dietary supplementation of extra virgin olive oil counteracted the damage effect of the pesticide by the enhancement of antioxidant defence system and the decline of the lipid peroxidation. Furthermore, the resistance to lipid peroxidation of erythrocyte membranes is modulated by both dietary fatty acid composition and antioxidant content. Consequently, the fatty acid composition of erythrocytes can be influenced by the type of dietary fat in rats.

## Methods

### Materials

**Figure 2:**
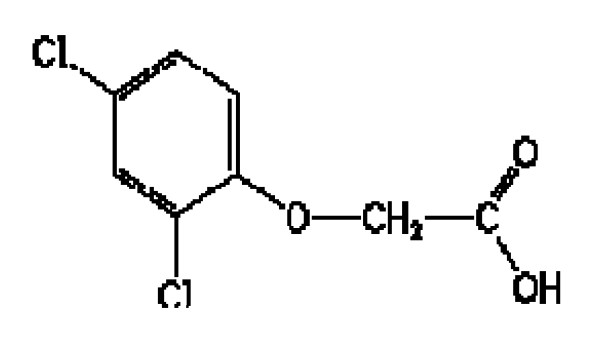
**Chemical structure of 2,4-D**.

2, 4-D commercial formulation (Désormone Lourd) consisting of 600 g/l 2,4-D Ester butylglycol, register number *H.96064 (SEPCM)*, available in Tunisia, was used in experimentation (Figure 2). 2-Thiobarbituric acid (TBA) was obtained from Sigma Chemicals Co (Taufkirchen, Germany). Folin-Ciocalteu phenol reagent was purchased from Fluka Biochemika (Buchs, Switzerland). All other used chemicals were of analytical grade and were obtained from Sigma Chemicals Co or Merck (Darmstadt, Germany).

### Oil sample analysis

The EVOO used in this experiment was obtained from Chétoui cultivar grown in the North of Tunisia. The OOHF were extracted from EVOO by Montedoro et al. (1992) method [59]. However, the OOLF was obtained from EVOO as follows: EVOO was homogenized for 1 min with water (1:1, v/v) and the oil was separated by centrifugation. This procedure was repeated six times. Then, the oil fraction (OOLF) was filtered through a cellulose acetate membrane.

The fatty acid methyl esters (FAME) were prepared by dissolving 0.1 g of EVOO or OOLF in 2 ml of heptane and 0.2 ml of KOH (0.2 N) in methanol and incubated for 1 hour. Chromatographic separation was carried out using a Hewlett-Packard (HP 5890) chromatograph (Hewlett-Packard Ca Palo Alto, Calif USA), a split/splitless injector, and a flame ionisation detector (FID) linked to an HP Chemstation integrator. A fused-silica capillary column, HP-Innowax (30 m × 0.25 mm × 0.25 µm), was used with nitrogen as the carrier gas at a flow rate of 1 ml/min; flame ionisation detection temperature 280°C; injector temperature 250°C; oven temperature programmed from 170 to 270°C at a rate of 5°C per min. Results were expressed as relative percent of total area [60].

Oxidation stability was evaluated by the Rancimat apparatus (Model 743, Metrohm Schweiz AG, Zofingen, Switzerland) using 3 g of oil sample heated to 120°C with 20 l/h air flow [61]. Stability was expressed as oxidation induction time (hours).

Carotenoids and chlorophylls (mg/kg oil) were determined at 470 and 670 nm, respectively, in cyclohexane using the specific extinction values according to the method of Minguez-Mosquera et al. [62].

The phenolic compounds were extracted, estimated colorimetrically at 765 nm using the Folin-Ciocalteau reagent, and expressed as hydroxytyrosol equivalents as reported by Montedoro et al. [59].

For the α-tocopherol analysis, the sample was diluted with *n*-hexane (1:10), the mixture was vortexed and 200 μl was transferred to a test tube containing 600 μl of methanol and 200 μl of internal standard (300 μg/ml). HPLC separation was carried out on a Hewlett-Packard system (Waldbronn, Germany) equipped with a HP-1100 pump, a Rheodyne model 7725 injector (Cotati, CA, USA, loop volume 20 μl), a HP-1200 M multi-array detector and a Supelcosil ODS-2 column (150 × 4.5 mm id., film thickness 5 μm) [63].

### Diets and Animal treatment

Male adult Wistar rats (Central Pharmacy, Tunisia), weighing about 200 to 230 g, were housed at 22 ± 3°C, with 12- hour light-dark periods, a 40% minimum relative humidity and free access to water and standard diet (protein 17%, carbohydrate 62%, lipids 4%, ash 7%, and moisture 10%) (SICO, Sfax Tunisia). All the breeding phases and experiments were conformable to the rules of the Tunisian Society for the Care and Use of Laboratory Animals. All experiments were conducted at the animal facilities of the faculty of Medicine, Monastir; with the approval of the Faculty of Medicine Ethics committee.

After acclimatization to the laboratory conditions for one week, the animals were divided into 8 groups of 10 animals each. Group C included the control animals and received 1 ml of distilled water gavages daily and fed with a standard diet. Group D was gavaged a daily dose of 2,4-D at a concentration of 5 mg/kg body weight (b.w.) and fed with the standard diet. Group D/EVOO was treated simultaneously with 2,4-D at a dose of 5 mg/kg b.w. and EVOO (300 μl) daily by gavage. Groups D/OOHF and D/OOLF received daily 5 mg/kg b.w. of 2,4-D followed by hydrophilic fraction (HF) supplementation (1 ml) and lipophylic fraction (LF) (300 μl) by gavage, respectively. The animals in EVOO, OOHF and OOLF groups were given EVOO (300 μl), hydrophilic fraction (1 ml; the same amount of phenols that we found in 300 μl of oil = 0.16 mg) and lipophilic fraction (300 μl), respectively.

Each group was kept on the treatment for 4 weeks. Water and food consumption and the individual animal body-weight were recorded daily throughout the experiment. At the end of the experimental period, the rats were kept fasting overnight and were sacrificed under diethyl ether anaesthesia.

### Blood collections

Blood was drawn by cardiac puncture and collected into evacuated tubes containing EDTA solution as anticoagulant for determination of haemoglobin (Hb) and heparin for dosage of antioxidant enzyme activities. Erythrocytes were separated from blood plasma by centrifugation (600 × *g*, 10 min) at 4°C and washed three times with cold phosphate-buffered saline (PBS; 150 mmol l^-1 ^NaCl, 1.9 mmol l^-1 ^NaH_2_PO_4_, 8.1 mmol^-1 ^Na_2_HPO_4_, pH 7.4 and stored at -80°C in aliquots until the analysis. The Haemoglobin (Hb) assay is based on the colorimetric cyanomethemoglobin method according to Drabkin [64].

### Biochemical analysis

Plasma malondialdehyde (MDA) levels were determined as described previously by Yagi [65]. 125 μl of serum were homogenized by sonication with 50 μl of TBS, 125 μl of TCA-BHT in order to precipitate proteins and then centrifuged (1000 g, 10 min, 4°C). 200 μl of supernatant were mixed with 40 μl of HCl (0.6 M) and 160 μl of TBA dissolved in Tris and the mixture was heated at 80°C for 10 min. The absorbance of the resultant supernatant was read at 530 nm. The TBA-rm amount was calculated using a 156 mM^-1 ^cm^-1 ^extinction coefficient.

Antioxidant enzyme activities were analyzed using a BioRad UV-Visible spectrophotometer with a "kinetics" program (BioRad, Mares la Coquette, France). The measurement of SOD, GPx and GR activities in erythrocytes were performed by the commercially available diagnostic kits supplied by Randox Laboratories. CAT activity was measured at 25°C according to Aebi's method [66], by measuring H_2_O_2 _concentration decrease at 240 nm.

Fatty acids were analyzed as fatty acid methyl esters (FAMEs) by gas chromatography analysis as previously described [67]. Briefly, total lipids were extracted from the erythrocytes by the modified method mentioned by Folch et al [68] using a chloroform-methanol (2:1, vol/vol) solvent system containing 0.01% butylated hydroxytoluene as antioxidant. Aliquots of the total lipids were converted into the methyl esters through a mixture of methanol-hexane-H_2_SO_4 _(75:25:1, vol/vol) as the methylation reagent, at 90°C for 90 min. FAMEs were analyzed in duplicate, and 1 μl of each sample was injected into the gas chromatography system (Hewlett Packard, Palo Alto, Calif.) equipped with a flame ionization detector and a polar fused silica capillary column HP-Innowax with cross-linked PEG, Carbowax 20 M (30 m × 0.25 mm ID and 0.25 μm as film thickness). The oven temperature was programmed to increase from 180°C to 250°C at a rate of 10°C/min, and the injector and detector temperatures were 220°C and 280°C, respectively. Fatty acid methyl esters were identified by comparing their retention times with those of individual standards.

### Statistical analysis

The data were analyzed using the Statistical Package for Social Sciences (SPSS) programme, release 11.0 for Windows (SPSS, Chicago, IL, USA). In each assay, the experimental data represent the mean of ten independent assays ± standard deviations. The results were analyzed by an ANOVA with a Bonferroni correction in order to perform multiple comparisons. Tukey's test was used to determine any significant differences between analytical parameters of supplemented diets. The statistical significance was set at *p *< 0.05.

## Abbreviations

CAT: catalase; EVOO: extra virgin olive oil; HB: Haemoglobin; GPX: glutathione peroxidase; GR: glutathione reductase; LDL: low density lipoprotein; MDA: malondialdehyde; MUFA: monounsaturated fatty acid; OOLF: olive oil lipophilic fraction; OOHF: olive oil hydrophilic fraction; PUFA: polyunsaturated fatty acid; UFA: unsaturated fatty acid; SFA: saturated fatty acid; SOD: superoxide dismutase; 2,4-D: 2,4-Dichlorophenoxiacetic acid.

## Competing interests

The authors declare that they have no competing interests.

## Authors' contributions

NA carried out the studies, acquired the data, performed the data analysis, drafted and revised the manuscript. TW played a major role in all the experimental procedures of this study. DS carried out olive oil analysis and provided technical assistance in the preparation of the manuscript and revised it. IM carried out olive oil analysis. KA was involved with the experimental work performed towards this manuscript. HM & AN involved in the design and organization of the study, interpreted the results and revised the manuscript.

All authors have read and approved the final manuscript.
